# The Synergistic Effects of Celecoxib and Sodium Valproate on Apoptosis and Invasiveness Behavior of Papillary Thyroid Cancer Cell Line *In-vitro*

**Published:** 2018

**Authors:** Maryam Fanian, Maedeh Bahmani, Mojdeh Mozafari, Samaneh Naderi, Marzieh Alizadeh Zareie, Mohammad Ali Okhovat, Jamileh Saberzadeh, Ali Dehshahri, Mohammad Ali Takhshid

**Affiliations:** a *Diagnostic Laboratory Sciences and Technology Research Center, School of Paramedical Sciences, Shiraz University of Medical Sciences, Shiraz, Iran. *; b *Department of Biotechnology, School of Pharmacy, Shiraz University of Medical Sciences, Shiraz, Iran.*

**Keywords:** Papillary thyroid cancer, Apoptosis, Invasion, Migration, Celecoxib, Sodium Valproate

## Abstract

Metastasis to lymph nodes and distant organs is the main challenge in the treatment of papillary thyroid cancer. In the current investigation, we aimed to evaluate the synergistic effects of celecoxib (CX) and sodium valproate (VPA) against cell survival, invasiveness properties, and expression of metalloproteinase-2 and -9 (MMP-2 and MMP-9) in papillary thyroid cancer cell line, B-CPAP cells. The effect of CX and VPA on B-CPAP cells viability and apoptosis were investigated using MTT assay and annexin V/7-AAD flowcytometry, respectively. The effects of the drugs on invasiveness properties of B-CPAP cells and expression of MMP-2 and MMP-9 were evaluated using transwell assay and real time PCR, respectively. MTT assay showed that CX and VPA decreased viability of B-CPAP cells dose dependently (IC_50_ 32.4µM and 6.8 mM, respectively). Combination of CX (5 μM) and VPA (2.5 and 5 mM) increased apoptosis, and reduced cell migration and invasion of B-CPAP cell, synergistically. Real time PCR results showed that both CX (5 µM) and VPA (2.5 and 5 mM) reduced MMP-2 expression (*P* < 0.05) but had no significant effects on the expression of MMP-9. Our findings suggest that CX and VPA synergistically increase apoptosis and suppress migration and invasion of B-CPAP cells through inhibition of MMP-2 expression.

## Introduction

Papillary thyroid cancer (PTC) is the most common type of thyroid cancer worldwide with increasing incidence annually (1). PTC is accurately diagnosed using fine needle aspiration biopsy of thyroid nodule, ultrasound imaging, and nuclear scan (2). Early diagnosis of PTC and using a combination of therapeutic modalities including surgical resection of tumor, radioiodine therapy, and thyroid hormone replacement is often associated with good clinical outcome and excellent prognosis of the disease (3). However, in 10 to 15% cases of PTC, metastasis to lymph nodes of the neck (4) or uncommonly to lung or bone (5) is occurred that limited the clinical outcome of therapy. Thus, more studies in order to understand the molecular pathways that involve in the proliferation and metastasis of PTC in order to find alternative therapeutic modalities against proliferation and especially metastasis of PTC are needed.

Cyclooxygenase2 (COX-2) is a rate limiting enzyme in the biosynthetic pathway of prostaglandins and thromboxanes. Increase expression of COX-2 and its association with cancer progression and metastasis has been shown for many tumors (6). In the case of PTC, increased expression of COX-2 is shown in the tumor tissue compared to normal thyroid tissue (7, 8). A significant associations between expression of COX-2, clinical stage (III-IV) and tumor diameter of PTC has also been demonstrated (9). Moreover, the role of COX-2 in the cervical lymph metastases of PTC has been proposed (10).

Histone deacetylases (HDACs) are a group of enzymes that catalyze the removal of acetyl group from amino group of lysine residues in variety proteins including histone proteins. Deacetylation of histones changes the organization of chromatin from the transcriptionally active form into a compact inactive heterochromatin form. Consequently, dysregulation of HDACs activity may cause aberrant expression of many genes including those involved in the control of cell proliferation and apoptosis (11). Increased expression of HDACs and its correlation with the several aspects of cancer such as un-controlled cell growth, angiogenesis, and metastasis has been demonstrated in many studies. In addition, the beneficial effects of HDAC inhibitors (HDACi) on the suppression of cell proliferation, induction of tumor cell apoptosis, and inhibition of metastasis have been demonstrated. In this context, the therapeutic efficacy of HDACi in inducing cell death of thyroid cancer cell lines has been reported (12).

Drug combination therapy is introduced as a kind of therapeutic intervention in which two or more drugs with different mechanism of action is concurrently used for the treatment of a disease. Increase in therapeutic effectiveness compares to single drug therapy, due to impacts of drugs on different aspects of the disease, and reduced in drugs side effects, due to use of lower concentrations of the drugs, are two main advantages of combination therapy. Valproic acid (VPA) is a HDACi with the ability to suppress the proliferation of several malignant cells, including thyroid cancer cells (13). Moreover, VPA has the ability to increase iodine transport into thyroid cells (14). Celecoxib (CX) is a selective inhibitor of COX-2 activity. The inhibitory effects of CX against the proliferation and metastasis of several tumors including thyroid cancers have been reported (15). 

Matrix metalloproteinase-2(MMP-2) and matrix metalloproteinase-9 (MMP-9) are zinc-dependent proteinases which facilitate metastasis of tumors cells via catalyzing degradation of extracellular matrix proteins. Increased expression of MMP-2 and MMP-9 in PTC (16) and furthermore the association of their serum levels with invasiveness of PTC have been demonstrated in previous studies (17). To the best of our knowledge, there is no previously report for the use of combined VPA+CX therapy against proliferation and metastatic potentials of PTC tumors cells. To this end, in the current investigation, we aimed to evaluate the synergistic effects of CX and VPA against cell survival, apoptosis, migration, and invasion of BCPAP cells and expression MMP-2 and -9 in these cells.

## Experimental

*Cell culture condition and drug treatment*

Human thyroid papillary carcinoma cell line, B-CPAP cell, was purchased from Pasture Institute, Iran (ACC 273). The cells were maintained in RPMI1640 supplemented with 10% of heat inactivated fetal bovine serum, 100 units/mL of penicillin, and 0.1 mg/mL of streptomycin (Sigma-Aldrich), at 37 °C, 5% CO2 incubator. Stock solutions of CX and VPA (Sigma-Aldrich) were prepared in dimethyl sulfoxide (DMSO) (Sigma-Aldrich). The Final concentration of DMSO in culture medium of all experimental groups including control group was 0.1% which had no significant effects on cell viability.

*Evaluation of the effects of drugs on cell viability using MTT assay*

The effect of the drugs on B-CPAP cell viability and proliferation were investigated using MTT assay. Briefly, 5 × 10^3^ of B-CPAP cells were seeded per well in 96-well plates. After 24 h, the cells were treated with different concentration of CX (5-60 µM) and VPA (1-10 mM). The lowest concentrations of the both drugs with significant lethal effect on the cells (5 µM for CX and 2.5 and 5 mM for VPA) were used for combination therapy. Forty-eight h after the treatments, MTT assay was done by addition of MTT solution [3-(4,5-dimethylthiazol-2-yl)-2, 5-diphenyltetrazolium bromide] to culture medium. The formed formazan was then solubilized using DMSO and the absorbance of solution was measured at 490 nm wavelength (18). 

*Determination of total apoptosis using flowcytometry*

To investigate the effects of CX and VPA on apoptosis of B-CPAP cells, B-CPAP cells were seeded in T25 flasks and treated with CX (5 µM), VPA (2.5 and 5.0 mM), and combination of both (5 µM of CX + 2.5 mM of VPA) for 48 h. The cells were washed in cold PBS and suspended in 1 mL of ice cold binding buffer. Annexin V (5 µL) and 7-AAD (5 µL) was then added to 100 µL of cell suspension , gently mixed, and incubated for 15 min in darkness at room temperature. The cells were analysed within 1 h using a flowcytometer analyser (BD FACS Calibur, BD Bioscince, USA). A total of 10,000 events were acquired for each sample. The percent of total apoptosis (early + late apoptotic cells) was calculated for each sample. 

*Migration and invasion assay*

To examine the effects of drugs on metastatic behavior of B-CPAP cells, migration and invasion assay were conducted using commercial 24-well transwell insert (8 µm pore filters, BD Bioscience, Bedford, MA) as previously described (19). Briefly, for migration assay the cells were treated with the drugs for 48 h and kept under starvation condition for 24 h. Five-thousand treated B-CPAP cells were then transferred to the upper part of each transwell, incubated at 37 °C, and allowed to migrat to the lower part of the transwell through a porous membrane for 24 h. The migrated cells under the lower surface of insert were fixed with methanol, stained using 0.5% crystal violet,and counted at five fields of lower power (400X) of a light microscope. Invasion assay was performed in the same way as migration assay except that the transwell insert porous membranes were precoated with 100 µL (1 mg/mL) of matrigel (BD Bioscience, Bedford, MA). 

*Evaluation of the VPA and CX effects on the expression of MMP-2 and MMP-9 using quantitative Real-time PCR*

MMP-2 and MMP-9 are key proteolytic enzymes involved in metastasis of a variety of tumors. An increase in expression of MMP-2 and MMP-9 at both mRNA and protein level and their correlation with malignant potential of PTC has been reported by several authors (16, 17). To investigate the effects of the drugs on expression of MMP-2 and MMP-9, B-CPAP cells were treated with the CX (5 µM), VPA (2.5 and 5.0 mM), and combination of both (5 µM of CX + 2.5 mM of VPA) for 48 h. The treated cells were then harvested and total RNA was extracted using TRIzol reagent (Thermo Fisher Scientific) and used for synthesis of cDNA using PrimeScript RT Reagent Kit (Takara, Japan). The relative expression of MMP-2 and MMP-9 were then measured in a Thermal Cycler (Bio-Rad, Waltham, MA, USA) using specific primers for MMP-2 and MMP-9 and SYBR PrimeScript mRNA quantitative real-time polymerase chain reaction Kit (Takara, Japan). Forward (F) and reverse (R) specific primer sequences for MMP-2 and MMP-9 were: MMP-2(F), 5′-TGGAGATACAATGAGGTGAAGAAG-3′; MMP-2(R), 5′-GAAGGCAGTGGAGAG GAA G-3′; MMP-9(F), 5′-TGACAGCGACAAGAAGTGG -3′; and MMP-9(R), 5′-GTGTGGTGGT GGTTGGAG -3′. Gene expression was normalized to the level of GAPDH within each sample using the relative ΔΔCT method. 

*Statistical analyses*

SPSS statistical program (version 15) was used to analyze the data. The data were represented as mean ± standard deviation (SD) of at least three independent experiments. The presences of significant difference between the groups were evaluated using one-way ANOVA followed by LSD post-hoc test. *P *< 0.05 was considered to be statistically significant. The IC_50_ values for CX and VPA in MTT assay experiments were calculated using the GraphPad Prism 5 (Version 5.01, GraphPad Software, Inc., USA).

## Results

*The effects of CX and VPA on viability of B-CPAP cell line*

**Figure 1:**
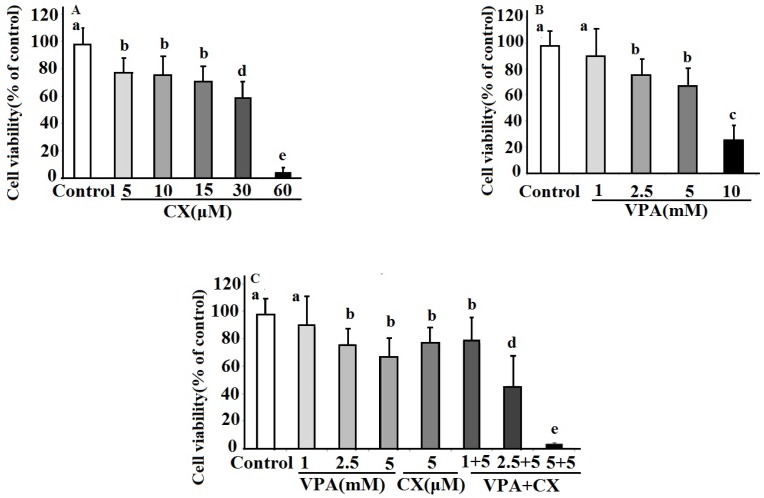
The effects celecoxib (CX), sodium valproate (VPA) and combination of both drugs (VPA+CX) on viability of BCPAP cells using MTT assay. The represented data are mean ± SD of at least three independent experiments and were analyzed using one-way ANOVA followed by Tukey’s post-hoc test. In each figure**, **groups indicated with different letters (a, b, c, d and e) had statistical significant differences at *P *< 0.05.

The effects of CX, VPA, and combination of both on viability of B-CPAP cells were show in Figure 1. As can be seen in the Figure 1A, CX dose dependently decreased viability of B-CPAP cells (IC_50_ = 32.4 µM). B-CPAP cells viability is also decreased in a dose dependent manner in VPA-treated cells (IC_50_ = 6.8 mM)(Figure 1B). Combination of both drug at all used concentration decreased cell viability. As illustrated in Figure 1C the combination of 2.5 and 5 mM of VPA plus 5 µM of CX showed significant more inhibitory effects on cell viability compared to use of each drug alone. The maximum effect was obtained at combination of 5 µM of CX plus 5 mM VPA, which decreased cell viability to about five percent of control untreated cells.

*The synergistic effects of VPA and CX on apoptosis of B-CPAP cells*

**Figure 2:**
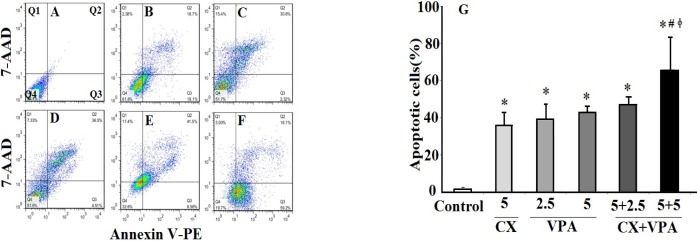
The effects of VPA and CX on apoptosis of B-CPAP cells. Represented flowcytometric charts (A-F) show the percentage of live (Q4), early apoptotic (Q3), late apoptotic (Q2), and necrotic cells (Q1) in different group (A: control group, B: 5 µM of CX, C: 2.5 mM of VPA, D: 5 mM of VPA , E: 5 µM of CX + 2.5 mM of VPA, F: 5 µM of CX + 5 mM of VPA). Histogram (G) compares the percentage of total apoptotic cells (Q2+Q3) in CX, VPA, and CX+VPA -treated and control B-CPAP cells. The represented data are mean ± SD of at least three independent experiments. Data were analyzed using one-way ANOVA followed by Tukey’s post-hoc test. ^*^P < 0.05 compared to control group; ^#^*P *< 0.05 compared to CX (5 µM) group, ^ɸ^*P *< 0.05 compared to VPA (2.5 and 5 mM)-treated groups.

Figure 2 shows the results of annexin V/7-AAD flowcytometric analyses (Figures 2A-E) of VPA and CX**-**induced apoptosis of B-CPAP cells. The percentage of total apoptotic cells (Q2+Q3) was increased by both CX (5 μM) and VPA (2.5 and 5 mM) compared to the control group. As shown in Figure 2G, co-treatment of CX (5 μM) with VPA (2.5 and 5 mM) increased the percentage of total apoptotic cells compared to CX-treated and VPA-treated group (2.5 and 5 mM) (***P*** < 0.05), suggesting synergistic effects of CX and VPA on inducing apoptosis of B-CPAP cells.

*Migration and invasion assay*

**Figure 3:**
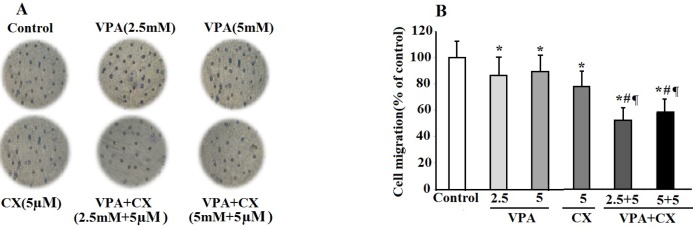
The effects of VPA and CX on the migration of B-CPAP cells *in-vitro*. (A) Representing light microscopy photomicrographs (40x) showing the migration ability of BCPAP cells in the control, VPA and CX-treated groups. (B) Histogram compares the mean ± SD of migrated cells in different group. Data was obtained from at least three independent experiments and were analyzed using one-way ANOVA followed by Tukey’s post-hoc test. ^*^P < 0.05 compared to control group; ^#^*P *< 0.01 compared to CX (5 µM); ^¶^*P *< 0.001 compared to VPA (2.5 and 5 mM)-treated cells.

To evaluate the possible synergistic effects of VPA and CX on metastatic potential of B-CPAP cells, *in-vitro* migration and invasion assay was performed using a 24-well transwell insert. As can be seen in Figure 3, VPA (2.5 and 5 mM) and CX (5 µM) decreased cell migration. The inhibitory effects of VPA (2.5 and 5 mM) and CX (5 µM) combination on cell migration were more significantly higher than the individual effects of each drug (*P* < 0.01). As illustrated in the histogram of Figure 3, the combination of VPA and CX reduced migration of B-CPAP cells to about 50% of untreated-cells(*P* < 0.001).

**Figure 4:**
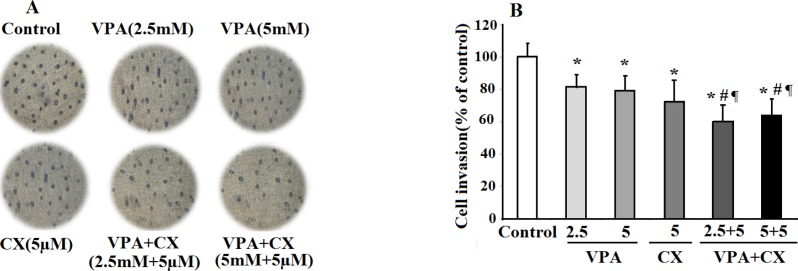
The effects of VPA and CX on the invasion of B-CPAP cells *in-vitro*. (A) Representing light microscopy photomicrographs (40x) showing the invasion ability of BCPAP cells in the control, drugs-treated groups. (B) Histogram compares the mean ± SD of invaded cells in different group. Data was obtained from at least three independent experiments and were analyzed using one-way ANOVA followed by Tukey’s post-hoc test. ^*^P < 0.05 compared to control group; ^#^*P *< 0.05 compared to CX (5 µM); ^¶^*P *< 0.01 compared to VPA (2.5 and 5 mM)-treated cells

To investigate the effects of drugs on invasiveness behavior of B-CPAP cells, the treated cells were transferred on matrigel coated membrane and allowed to invade the gel for 24 h. As can be seen in the photomicrographs of Figure 4A, treatment with the both CX (5 µM) and VAP (2.5, 5 mM) reduced the number of invaded cells compared to the control group. The maximum effect was obtained when the cells were treated with the combination of VPA and CX, indicating synergistic effects of these drugs in inhibiting invasion ability of B-CPAP cells. The results of statistical analyses were summerized in Figure 4B. As illusterated in the histogram the percent of invaded cells was significantly reduced to 82.04%, 78.90%, 72.15%, 60.14%, and 64.02% of control non-treated cells after treatment with VPA 2.5 mM, VPA 5 mM , CX 5 µM, VAP+CX (2.5 mM + 5 µM), and VAP+CX (5 mM + 5 µM), respectively.

*The effects of VPA and CX on the expression of MMP-2 and MMP-9*

**Figure 5:**
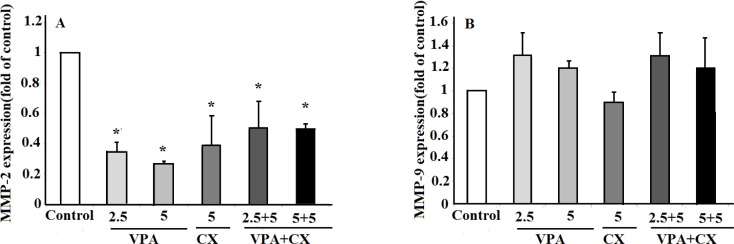
(A) The effects of VPA and CX on the exoression of MMP-2 and (B) MMP-9 in B-CPAP cells. Gene expression was normalized to the level of GAPDH within each sample using the relative ΔΔCT method. The represented data are mean ± SD of at least three independent experiments and were analyzed using one-way ANOVA followed by Tukey’s post-hoc test. ^*^P < 0.05 compared to control group.

Real time PCR was used to evaluate the effects of VPA and CX on the expression of MMP-2 and MMP-9. Our finding showed that both CX (5 µM) and VPA (2.5 and 5 mM) reduced the expression of MMP-2 expression significantly (*P* < 0.05). Treatment of B-CPAP cells with combination of CX (5 µM) and VPA (2.5 and 5 mM) was also reduced the expression of MMP-2 significantly (*P* < 0.05); however, no significant difference was observed between the combination therapy and when the drugs used individually (Figure 5A). No significant effects on the expression of MMP-9 were observed after treating B-CPAP cell with the drugs (Figure 5B). 

## Discussion

Due to role of metastasis in the poor outcome and mortality rate of PTC patients, finding of an efficient therapeutic modality that strongly inhibits growth and invasiveness characteristics of PTC cells is necessary. Combination therapy could be beneficial in this issue. In the present study, we evaluated the synergistic effects of CX and VPA on cell viability and invasiveness behaviors of B-CPAP cells. The first finding of our present study was the synergistic effects of CX and VPA on inducing apoptosis and decreasing viability of B-CPAP cells. The second finding was the synergistic effects of CX and VPA on inhibition of invasion and migration of B-CPAP cells in Transwell assay. Finally, we found that CX and VPA reduced the expression of MMP-2 at transcriptional level while had no significant effects on the expression of MMP-9.

Findings of MTT assay in this study revealed a decrease in viability of B-CPAP cells following treatment with VPA in dose and time dependent manner which consistent with the findings of previous studies conducted on other tumor cell lines (20). The lowest effective dose of VPA was 2.5 mM after treatment for 48 h. Flowcytometric analyses showed that VPA at 2.5 mM concentration induced apoptosis of B-CPAP cells, considerably. These results indicates that apoptosis is the main causes of VPA-induced cell death. In supporting our data, apoptotic effects of other HDACi such as trichostatin A and vorinostat have been demonstrated in previous studies (21). Although the exact molecular mechanisms responsible for the apoptotic effects of HDACi remain elusive yet, up regulation of miR-129-5p expression (12), increase in reactive oxygen species production (22), inhibition of cell survival signaling pathways such as RAS/RAF/ERK and PI3K/AKT /mTOR (23), and causing DNA double strand break DNA are among proposed mechanisms (23). 

Anti-cancer effects of selective COX-2 inhibitors including CX against numerous tumors have also been reported (24). Our data in the present study showed that CX reduced B-CPAP cell viability in the MTT assay dose dependently, with the lowest effective dose of 5 µM. At this concentration, CX increased cell apoptosis to about 25 folds of non-treated cells. Results also revealed a significant inhibition of B-CPAP cells migration and invasion at 5 µM concentration of CX. A significant down regulation of MMP-2 was also observed at this concentration. These findings are consistent with the results of previous reports which revealed an increased expression of COX-2 and its association with increased invasiveness potential of PTC tumor cells (25-26) and also with the effects of another COX-2 inhibitors, NS-398, on suppression of PTC cells migration and invasion (27).

Despite the beneficial effects of CX in the treatment of cancer, the major problem in the use of this drug is its side effects on cardiovascular system (28). Thus, the use of CX in low concentrations and concurrent with other anti-cancer agents in combination therapy procedures has been suggested. In this context beneficial effects of CX in improving therapeutic efficacy of chemotherapeutic agents (29); tyrosine kinase inhibitors (30), radiotherapy (31), and gene therapy (32) have been demonstrated. Combination therapy using HDACi has also considered as an attractive therapeutic strategy for the treatment of cancer in recent years (33). The synergistic effects of HDACi with a variety of therapeutic modalities including radiotherapy (34) and chemotherapy (35) have been demonstrated previously. Cha *et al.* demonstrated synergistic effects of VPA and tumor necrosis factor-related apoptosis-inducing ligand in inducing apoptotic cell death of PTC cells (13). In addition the synergy between HDACi and inhibition of NF-κB on proliferation of B-CPAP cells has also been demonstrated (36). 

In the present study, we conducted a combination therapy study using low concentrations of VPA and CX on cell survival and invasiveness properties of B-CPAP cell. Findings showed synergistic effects of this combination on cell death and apoptosis of B-CPAP cells. Furthermore, concurrent use of VPA and CX, decreased cell migration and invasion of B-CPAP cells compared to use of each drug alone. These findings are consistent with the observed synergistic effects of VPA and CX combination therapy against cell viability of neuroblastoma cells (37). The exact molecular mechanisms that are behind the synergistic effects of VPA and CX in B-CPAP cells viability remain unclear. It has been reported that VPA and other HDACi suppressed the IL-1beta- induced COX-2 gene expression and protein expression in the endometriosis (38). Thus, the observed synergistic effect between VPA and CX in our study might be due to their combined influences on COX-2 activity. In addition, it has been reported that both VPA and CX induced apoptosis of neuroblastome cells via up-regulation of Bax apoptotic protein and increase in Bax/Bcl2 ratio (37). Further studies are required to explore the role such mechanisms in the synergistic effects of VPA and CX in B-CPAP cells.

While both of the drugs decreased the expression of MMP-2 alone, the result did not reveal any synergistic interaction between CX and VPA against MMP-2 expression when used in combination. Although the definitive explanation for this observation is not yet available, the stimulatory effects of VPA on a signaling pathway by which CX reduces MMP-2 expression may be a possible description for this finding. Wnt/β-catenin signaling pathway may be a good candidate in this issue. In this signaling pathway, free cytoplasmic β-catenin translocates into the nucleus and activates transcription of MMP-2 gene (39). It has been demonstrated that CX reduced MMP-2 expression through inhibition of β-catenin accumulation in the nucleus (40). In contrary to CX, VPA activates Wnt/β-catenin signaling pathway (41). Taken together, it may hypothesize that VPA has two activities, on one hand it reduced MMP-2 expression and on the other hand it antagonize CX effect on MMP-2 expression. Further studies are necessary to elucidate the details of CX and VPA mechanisms in this issue. 

 In the present study, annexin V/7-AAD detection flow cytometric technique was applied as a reliable technique for accurate quantifying cell apoptosis. However, the mechanism of apoptotic effects of VPA and CX did not investigate, therefore, additional techniques for apoptosis assay such as analyses of caspases activation, determination of Bax/Bcl2 ratio, and DNA fragmentation assay are required to determine the mechanism underlying apoptotic effects of VPA and CX(42). Moreover, the results of real time PCR showed a decreased level of MMP-2 upon drugs treatment, suggesting that VPA and CX probably affects B-CPAP invasiveness through inhibition of MMP-2. Further studies employing gelatin zymoraphy or direct measuring of MMPs activity and assay for MMPs inhibitor (43) are suggested to explore the exact effects of VPA and CX on MMPs activity.

In conclusion our findings in this study suggest that CX and VPA could act synergistically at low concentration to increase B-CPAP cell death through induction of apoptosis. In addition, they have ability to reduce cell migration and invasion through inhibition of MMP-2 expression.
